# Effect of a 980-nm diode laser on post-operative pain after endodontic treatment in teeth with apical periodontitis: a randomized clinical trial

**DOI:** 10.1186/s12903-021-01401-w

**Published:** 2021-01-22

**Authors:** Tuna Kaplan, Güzide Pelin Sezgin, Sema Sönmez Kaplan

**Affiliations:** Department of Endodontics, Faculty of Dentistry, Biruni University, Istanbul, Turkey

**Keywords:** Diode laser, Irrigation, Post-operative pain, Root canal treatment, Visual analogue scale

## Abstract

**Background:**

This study aimed to assess the effect of a 980-nm diode laser following chemomechanical root canal preparation on the severity of post-operative pain (PP) after root canal treatment (RCT).

**Methods:**

This study included asymptomatic, single-rooted teeth with a periapical index (PAI) score of 3 or 4. All the patients received RCT, including dressing with calcium hydroxide (Ca(OH)_2_), during two visits. The patients were randomly divided into two groups (*n*:30): a control group, in which the final irrigation was performed using 5 ml of 2.5% sodium hypochlorite (NaOCI), followed by 5 ml of 17% and ethylenediaminetetraacetic acid (EDTA) and 5 ml of distilled water, and a laser group, in which the root canals were irradiated using a 980-nm diode laser after the final irrigation at both visits. The pain levels after both visits were evaluated using a visual analogue scale (VAS) after 8 h, 24 h, 48 h and 7 d. In addition, the time intervals to medication intake were recorded. The collected data were statistically analysed using the chi-square and Mann–Whitney *U* test (*p* < 0.05).

**Results:**

The average pain level in the control group 24 h after the first visit was significantly higher than that in the laser group (*p* < 0.05). The average pain level 24 h and 48 h after the second visit was significantly higher in the control group (*p* < 0.05). The levels of PP 24 h after the first visit were higher than those after the second visit only in the control group (*p* < 0.05). After the first visit, analgesic use in the control group was significantly higher after 8 h (40%) and 24 h (23%) as compared with that in the laser group (*p* < 0.05).

**Conclusions:**

Root canal irradiation with a diode laser may reduce PP after RCT in single-rooted teeth with a PAI score of 3 or 4.

*Trial registration*: Effect of the Diode Laser on Post-operative Pain After Endodontic Treatment in Teeth with Apical Periodontitis: NCT04486196. Registered 24 July 2020—Retrospectively registered, http://clinicaltrials.gov/ct2/show/NCT04486196

## Background

Post-operative pain (PP), which causes discomfort, is a frequent occurrence after root canal treatment (RCT). Upon completion of RCT, PP has been reported to vary between 3 and 58% and to be observed in up to 12% of patients within 24 to 48 h of the treatment, according to a visual analogue scale (VAS) [1, 2]. The prevention of PP is important to ensure patient comfort [3]. Many factors, including mechanical, chemical and microbial, can cause pain after endodontic procedures, whether root canal obturation or intra-canal medicament application. The aetiology of PP is primarily associated with the extrusion of microorganisms and their products to the periapical area via over-instrumentation or irrigation solutions [4, 5].

In general endodontic practice, sodium hypochlorite (NaOCl) is the most frequently used irrigation solution due to its extensive antimicrobial activity and ability to dissolve organic material [6]. Nevertheless, due to its limited penetration capability caused by inadequate irrigation dynamics, NaOCl may not always eliminate microorganisms in difficult to reach areas, including dentinal tubules of root canals [7, 8]. Thus, the advice is to use demineralizing agents as adjuvants in endodontic therapy. According to previous research, opening dentinal tubules might lead to improved canal disinfection by facilitating NaOCl penetration into dentinal tubules [9]. A number of studies have advised the combined application of NaOCl and ethylenediaminetetraacetic acid (EDTA) for elimination of the smear layer [10, 11], with this combination been demonstrated to be more effective in terms of disinfection than NaOCl alone [12].

The risk of extrusion of irrigants is high among teeth with a physiologically wide apical foramen or damaged apical foramen due to iatrogenic errors. Irrigants with strong cell toxicity that extrude into periapical tissues can lead to PP and even tissue necrosis [13]. However, according to previous research, microorganisms are the most common cause of PP [14]. A previous research also suggested that microbial removal in the root canal system might be limited by the morphological complexity (e.g. different dimensions) of the root canal system restricting penetration of irrigation solutions beyond the main canal. Such differences compromise canal debridement [15].

The use of diode lasers, in addition to conventional endodontic therapy, has recently been proposed in RCT [16]. Various researchers have observed effective disinfection of the root canal by diode laser irradiation [17–19]. Due to the large water transmission capacities of diode lasers (810, 940 and 980-nm wavelengths), they can reach bacteria in deeper layers of dentinal tubules [20]. Schoop et al. [21] reported that using a 980-nm diode laser resulted in changes in dentinal surfaces and an increased bactericidal effect. Other studies noted that laser irradiation appeared to decrease PP after RCT [22, 23].

As microorganisms are the most common cause of PP, the present study aimed to evaluate the effect of the application of a 980-nm diode laser following conventional irrigation on the severity of PP in asymptomatic single-rooted teeth with a periapical index (PAI) score of 3 or 4. To the best of our knowledge, there are no studies in the literature with a similar study design. The null hypothesis of the present study was that there would be no significant difference in PP levels between a laser and control group.

## Methods

This study is a parallel randomized controlled trial, with an allocation ratio of 1:1. The study followed the CONSORT guidelines (Additional file 1) and was approved by the ethics committee of Biruni University (2015-KAEK-43–19-04). All the patients read and signed an informed consent form containing details about the study, as well as the benefits and risks of the therapy.

### Sample size calculation

The sample size was calculated based on data obtained from a pilot study using G*Power 3.1 (Heinrich Heine University, Dusseldorf, Germany) software. The main research protocol was the same as that of the pilot study. The power calculation showed that the smallest sample size for each group was 25 patients, following these input conditions: effect size of 0.82, power of 80% and significance level of 0.05. Due to the probability of dropouts during the treatment or follow-up stages, 30 patients were included in each group, resulting in 60 patients in total in the present study.

### Patient selection and randomization

**Fig. 1:**
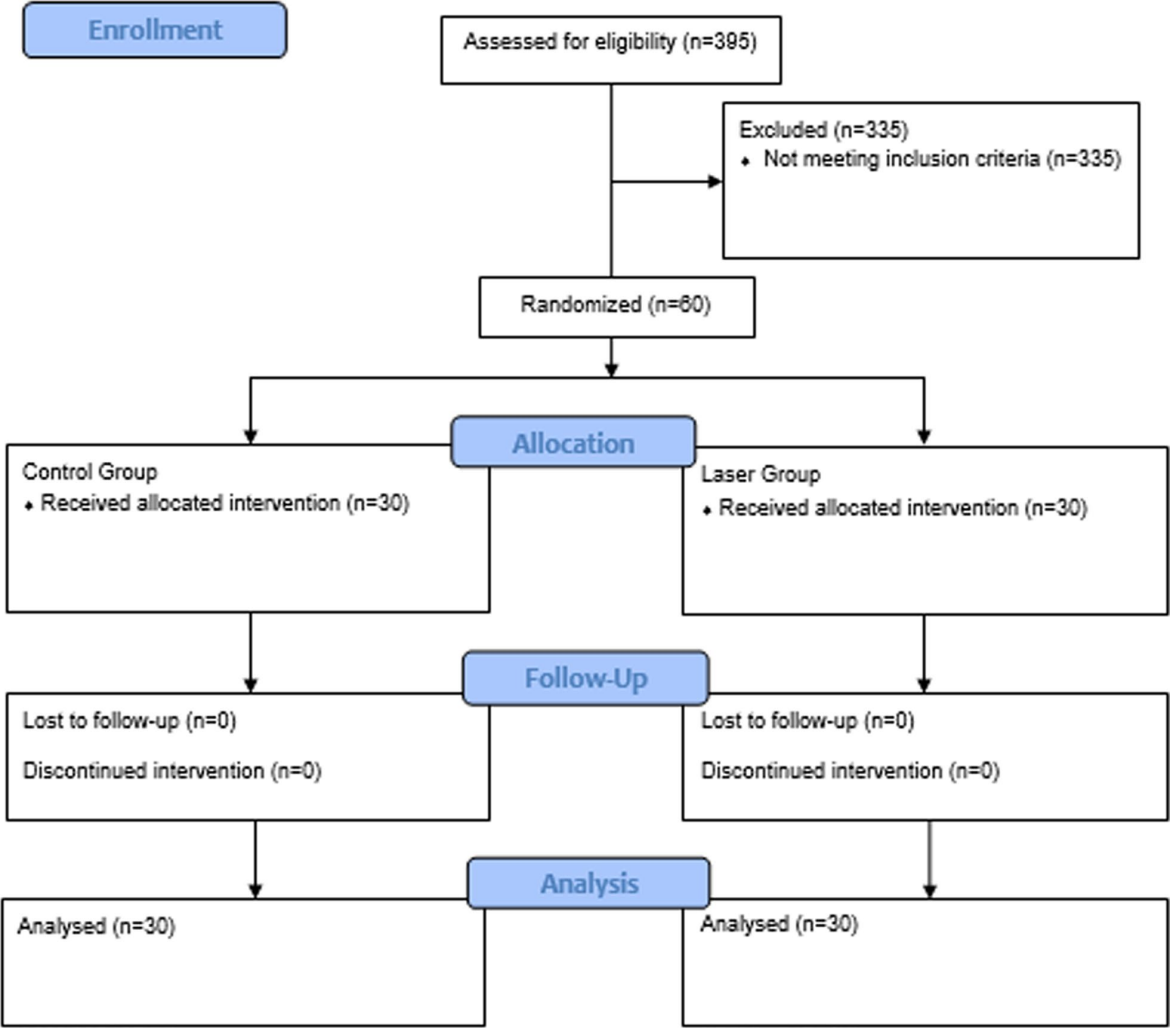
CONSORT 2010 Flow Diagram for randomized clinical trials

We examined 395 patients aged 18–65 y who were referred to the endodontics department of the faculty of dentistry of Biruni University. In total, 60 healthy patients who met the inclusion criteria were selected. Only patients who had asymptomatic, single-rooted teeth with a PAI score of 3 or 4 were included in this study. For diagnosis, both clinical and radiographic examinations were performed. To determine the periapical status, both panoramic radiographs (Sirona, Bensheim, Germany) and periapical radiographs (Dürr Dental, Bietigheim-Bissingen, Germany) obtained taken using the parallel technique were examined. Experienced radiology technicians took the radiographs. The exclusion criteria were antibiotic use within the last month, anti-inflammatory analgesic use within the last 5 d, systemic disorders, pregnancy or lactation, traumatic occlusions, the presence of other teeth requiring RCT, teeth with root canal fillings, calcified canals, root resorption, periodontal diseases or sinus tracts and severe crown destruction preventing rubber-dam application. The same operator performed all the endodontic treatment procedures over a period of 5 mos. To ensure randomized allocation before the RCT, a dental student blinded to the research process allocated the patients by asking each patient to select one of two sealed envelopes, which contained the group allocation code. In result, 60 patients were divided into two separate groups according to the root canal disinfection procedure: a control group and a laser group. A diagrammatical representation of the trial according to CONSORT is provided in Fig. 1.

### Treatment protocol

After local anaesthetic (4% articaine with 1:100,000 epinephrine) application and rubber-dam placement, the operator removed all former coronal restorations and caries present. Following access cavity preparation, the working lengths were determined electronically using a Propex Pixi device (Dentsply Maillefer, Ballaigues, Switzerland) and confirmed by periapical radiographs. The root canals were prepared using ProTaper Next nickel-titanium files (Dentsply Maillefer, Ballaigues, Switzerland) with an X-Smart Plus Endo Motor (Dentsply Maillefer, Ballaigues, Switzerland) at a speed of 300 rpm and 2 N/cm according to the manufacturer’s instructions up to the size of X4 (size 40, 0.06 taper). During root canal preparation, the canals were irrigated with 2 ml of 2.5% NaOCl using a 30-gauge, side-opening needle (Canal Clean; Biodent, Seoul, South Korea) positioned 3 mm short of the working length.

### Control group

The final irrigation was performed using 5 ml of 2.5% NaOCI, followed by 5 ml of 17% EDTA for 3 min and 5 ml of distilled water. The canals were then dried with paper points, and calcium hydroxide (Ca(OH)_2_) paste (Calsin, Karabağlar, Izmir, Turkey) was applied as intra-canal medicament. Subsequently, a temporary restorative material (Cavit-G; 3 M ESPE, St Paul, MN) was used to seal the access cavity. At the time of the first visit, all the patients were given a VAS (Additional file 2) to rate their PP. A second appointment was scheduled for 7 d later.

At the second appointment, the Ca(OH)_2_ was removed from the root canals with final irrigation and using an X4 file. The root canals were then obturated using the cold lateral condensation method with gutta-percha cones and AH Plus sealer (Dentsply Maillefer, Ballaigues, Switzerland). The coronal restoration was finished using a resin composite (Filtek Z250, 3 M ESPE, St. Paul, MN). The operator contacted the patients at pre-arranged times and asked about PP levels and analgesic intake.

### Laser group

The final irrigation was performed as in the control group, and the canals were dried with paper-points. During the laser treatment, both the operator and patient wore protective eyewear. Laser irradiation was applied using a 980-nm diode laser (Medency Primo 10 W Diode Laser, Vicenza, Italy) coupled with a 200-µm optical fibre (spot size of 0.02 cm in diameter and an area of 0.000314 cm^2^). The settings were as follows: output power of 2.4 W and energy of 12 J (each cycle) in the pulsed mode (pulse duration: 20 µs), irradiation for 10 s, followed by a 10-s pause, which comprised one cycle. This cycle was applied four times to each root canal. The average power used was 1.2 W (average power density = 3822 W/cm^2^) with a low frequency of 50 Hz. The optical fibre tip (length of 25 mm) was inserted at the working length. The root canals were then slowly (at a speed of 2 mm/s) irradiated from the apical to coronal using a continuous circling movement touching the dentinal walls in one cycle for each power. Following the irradiation procedure, Ca(OH)_2_ paste was applied as intra-canal medicament, and the access cavity was sealed using with a temporary restorative material, as in the control group. A VAS form (Additional file 2) was given to each patient to rate PP, and a second appointment was scheduled for 7 d later.

At the second appointment, the root canals were dried after Ca(OH)_2_ removal as in the control group. Afterwards, laser irradiation was performed as in the first appointment. Subsequently, the root canals were filled, and permanent restoration was performed as in the control group. At scheduled times, the patients were contacted to obtain information about PP and analgesic intake. This information was recorded on the VAS form (Additional file 3) by the operator.

### Patient questionnaire

The patients received training on how to complete the VAS form (Additional file 2) at home. The questionnaire assessed pain perception and the frequency of analgesic use after the first visit. None of the patients were prescribed medications immediately after the RCT. The patients completed the questionnaire 8 h, 24 h, 48 h and 7 d post-operatively. The pain level was categorized as none (0), mild (1–3), moderate (4–7) and severe (8–10). The patients were advised to take 600 mg of ibuprofen every 6 h for pain alleviation if they felt severe pain at any point during the follow-up period. Each patient recorded the time interval to medication intake. In addition, the age and sex of the patients were documented.

### Statistical analysis

All statistical analyses were performed using SPSS version 22 software (IBM SPSS, Turkey). A value of *p* < 0.05 was accepted as statistically significant. The Mann–Whitney *U* test was used for comparison of values at the different time points between the groups, and the Wilcoxon signed-rank test was used for within-group comparisons among the different time points. The Student’s *t* test was used to evaluate the age and sex distribution in the groups. For statistical analysis of differences in analgesic use, Fischer’s exact chi-square test was applied.

## Results

The demographic distribution of the patients is shown in Table 1. There were no significant differences in terms of the mean age and sex in the control and laser groups (*p* = 0.39, *p* = 1.00; Table 1).

**Table 1:** Demographic data of patients in control and laser groups

	Control	Laser	*p*
Age Mean ± SD	32.07 ± 10.54	34.43 ± 11.04	0.399^a^
Sex n(%)			
Male	10 (33.3)	10 (33.3)	1.000^b^
Female	20 (66.7)	20 (66.7)	

^a^Student t test

^b^Continuity (yates) correction

Table 2 shows the PP levels in the two groups after the first and second appointments. There was no significant between-group difference in PP levels 8 h after the first or second appointments (*p* = 0.076, *p* = 0.57). The pain level 24 h post-treatment in the control group was significantly higher than that in the laser group after both appointments (*p* = 0.002, *p* = 0.040). The pain level after 48 h in the control group was significantly higher than that in the laser group only after the second appointment (*p* = 0.040). There was no report of PP in either group after 7 d.

**Table 2:** Pain level distribution in the control and laser groups at 8 h, 24 h, 48 h and 7 d after treatment, both for the first and second visits

Pain level		1st visit			2nd visit		
		Control group	Laser group	*p*	Control group	Laser group	*p*
8 h	None	13 (43.3%)	14 (46.7%)		23 (76.7%)	24 (80%)	
	Mild	6 (20%)	16 (53.3%)		6 (20%)	6 (20%)	
	Moderate	8 (26.7%)	0 (0%)		1 (3.3%)	0 (0%)	
	Severe	3 (10%)	0 (0%)		0 (0%)	0 (0%)	
	Mean ± SD (median)	2.57 ± 2.85 (2)	1.0 ± 1.02 (1)	0.076	0.57 ± 1.13 (0)	0.3 ± 0.65 (0)	0.579
24 h	None	15 (50%)	26 (86.7%)		26 (86.7%)	30 (100%)	
	Mild	8 (26.7%)	4 (13.3%)		3 (10%)	0 (0%)	
	Moderate	7 (23.3%)	0 (0%)		1 (3.3%)	0 (0%)	
	Mean ± SD (median)	1.9 ± 2.3 (0.5)	0.33 ± 0.92 (0)	0.002*	0.3 ± 0.88 (0)	0 ± 0 (0)	0.040*
48 h	None	23 (76.7%)	27 (90%)		26 (86.7%)	30 (100%)	
	Mild	3 (10%)	3 (10%)		4 (13.3%)	0 (0%)	
	Moderate	4 (13.3%)	0 (0%)		0 (0%)	0 (0%)	
	Mean ± SD (median)	0.9 ± 1.84 (0)	0.2 ± 0.61 (0)	0.132	0.27 ± 0.69 (0)	0 ± 0 (0)	0.040*
7 d	None	30 (100%)	30 (100%)		30 (100%)	30 (100%)	
	Mean ± SD (median)	0 ± 0 (0)	0 ± 0 (0)	1.000	0 ± 0 (0)	0 ± 0 (0)	1.000

Mann Whitney U Test

^*^p < 0.05

The results of the comparison of PP levels after the first and second appointments are shown in Table 3. In both the control and laser groups, the levels of PP 8 h after the first appointment were significantly higher than those 8 h after the second appointment (*p* = 0.002, *p* = 0.017). The levels of PP 24 h after the first appointment were higher than those 24 h after the second appointment only in the control group (*p* = 0.005). No optical fibre breakage was observed during laser irradiation procedures.

**Table 3:** Comparison of pain levels after first and second visits separately in control and laser groups

Pain level		Control group			Laser group		
		1st visit	2nd visit	*p*	1st visit	2nd visit	p
8 h	None	13 (43.3%)	23 (76.7%)		14 (46.7%)	24 (80%)	
	Mild	6 (20%)	6 (20%)		16 (53.3%)	6 (20%)	
	Moderate	8 (26.7%)	1 (3.3%)		0 (0%)	0 (0%)	
	Severe	3 (10%)	0 (0%)		0 (0%)	0 (0%)	
	Mean ± SD (median)	2.57 ± 2.85 (2)	0.57 ± 1.13 (0)	0.002*	1.0 ± 1.02 (1)	0.3 ± 0.65 (0)	0.017*
24 h	None	15 (50%)	26 (86.7%)		26 (86.7%)	30 (100%)	
	Mild	8 (26.7%)	3 (10%)		4 (13.3%)	0 (0%)	
	Moderate	7 (23.3%)	1 (3.3%)		0 (0%)	0 (0%)	
	Mean ± SD (median)	1.9 ± 2.3 (0.5)	0.3 ± 0.88 (0)	0.005*	0.33 ± 0.92 (0)	0 ± 0 (0)	0.059
48 h	None	23 (76.7%)	26 (86.7%)		27 (90%)	30 (100%)	
	Mild	3 (10%)	4 (13.3%)		3 (10%)	0 (0%)	
	Moderate	4 (13.3%)	0 (0%)		0 (0%)	0 (0%)	
	Mean ± SD (median)	0.9 ± 1.84 (0)	0.27 ± 0.69 (0)	0.119	0.2 ± 0.61 (0)	0 ± 0 (0)	0.083
7 d	None	30 (100%)	30 (100%)		30 (100%)	30 (100%)	
	Mean ± SD (median)	0 ± 0 (0)	0 ± 0 (0)	1.000	0 ± 0 (0)	0 ± 0 (0)	1.000

Wilcoxon signed-rank test

^*^p < 0.05

Table 4 provides information on analgesic use in the two groups after the first and second appointments. After the first appointment, analgesic use was significantly higher after 8 h (40%) and 24 h (23%) in the control group as compared with that in the laser group (*p* = 0.000, *p* = 0.011).

**Table 4:** Comparison of groups in relation to analgesic use after first and second visits

Analgesic use	1st visit			2nd visit		
	Control group	Laser group	*p*	Control group	Laser group	*p*
8 h	12 (40%)	0 (0%)	0.000*	2 (6.7%)	0 (0%)	0.492
24 h	7 (23.3%)	0 (0%)	0.011*	0 (0%)	0 (0%)	–
48 h	4 (13.3%)	0 (0%)	0.112	0 (0%)	0 (0%)	–
7 d	0 (0%)	0 (0%)	–	0 (0%)	0 (0%)	–

Fisher’s Exact Test

^*^p < 0.05

## Discussion

After RCT, PP is common both in necrotic teeth and in teeth with periapical lesions. The most frequent causes of PP after RCT are microorganisms, followed by root canal preparation issues, such as over-instrumentation and inadequate shaping or irrigation, resulting in insufficient antimicrobial action during biomechanical procedures. As a result, keeping all the endodontic procedures limited within the root canal is very important to limit PP. Discord between measurements of the working length using radiographc and electronic methods is an additional problem [24]. Thus, in the present study, we used periapical radiographs and electronic apex locators in combination to obtain more accurate working length measurements. Even after adequate cleaning and shaping of root canals, patients may still experience PP, with pain thresholds of patients playing a role in PP sensations [3, 25–27]. This study aimed to evaluate the effect of the application of a diode laser following final irrigation on PP levels after RCT that took place during two visits.

In the present study, the study population was limited to healthy patients without any systemic disorders to eliminate PP risks as much as possible. Patients who had asymptomatic single-rooted teeth with a PAI score of 3 or 4 and no previous pain history were selected to eliminate potential pre-existing conditions that could contribute to PP. Patients at risk of experiencing reflective pain were not included, as pain in another tooth can affect the levels of PP caused by an operated tooth [28]. Tuner et al. [29] demonstrated that the risk of PP after conventional RCT was increased among patients with chronic diseases. Thus, the present study included patients with a PAI score of 3 or 4 (i.e. chronic disease), as such patients have an increased risk of PP. In similar studies, patients with single-rooted teeth with vital or necrotic pulp or failed endodontic treatments were selected to examine PP, excluding patients with medical disorders [3, 30, 31].

In some PP studies, the RCT was completed in a single visit both in necrotic and retreatment cases [23, 31, 32]. Similar to the present study, RCT was completed in two visits in other studies [33, 34]. However, these previous studies evaluated PP levels only after the first visit. In contrast, we evaluated pain levels both after the first and second visits to distinguish the effects of disinfection and obturation procedures on PP considering root canal obturation procedures might also be a cause of PP.

Many scales and methods can measure PP. The present study used the VAS for the assessment of PP, with values ranging from 0–10. This scale is easily understandable by patients and provides reliable, clear and valid results when used appropriately [35]. In many previous studies, the VAS form was used to evaluate PP in endodontics [33, 36, 37]. In the present study, the operator explained the nature of the scale clearly to the participants before the treatment to ensure accurate recordings of PP.

Various laser types are used in different fields of dentistry [38]. In endodontics, diode lasers are commonly applied for disinfection of the root canal system [39]. Diode lasers have considerable advantages, such as compactness, adaptability, ease of use and affordability [38, 39]. Although many studies have focused on the utility of laser therapy on PP in endodontics [23, 31, 34], the mechanism by which diode lasers may decrease PP remains a matter of debate. Some previous studies proposed that diode lasers ameliorated chronic pain by inducing anti-inflammatory activity [40, 41]. Gutknecht et al. [19] and Garcez et al. [42] found that the use of laser irradiation in infected root canals significantly decreased microbial numbers. Morsy et al. [30] also concluded that the strong antibacterial effect of diode lasers reduced PP. Thus, in the present study, we used a diode laser following conventional irrigation in necrotic teeth at the time of both the first and second appointments.

Diode lasers exert an antimicrobial effect mostly by thermal action [43]. In this study, similar to previous studies [44, 45], intra-canal laser irradiation was performed using a pulsed mode with circular movements to reduce heating of dentin, thereby not damaging the surrounding periodontal tissue. The canal wall temperature immediately decreases when a laser coupled with an activated 200-µm fibre optic tip is applied quickly from the apical to coronal direction. Hence, the tissues surrounding the tooth are only marginally affected, and periradicular tissues is not injured [46]. No adverse effects related to heating, were observed during laser irradiation in present study.

Previous studies reported different results regarding the effect of age and gender on PP [47, 48]. Although Ali et al. [47] found that age affected PP, Ng et al. [48] concluded that it did not have a critical effect. In the present study, we used a simple randomization method to divide the patients into two groups. According to this method, there were no statistically differences in terms of the mean age or sex between the laser and control groups. The absence of age- and sex-related differences increases the reliability of the study results.

After endodontic procedures, PP generally occurs during the first 2–3 d and decreases over time [23, 49]. Likewise, as shown by the results of the present study, after the first and second visits in both groups, PP was most prevalent after 24 h, with the patients declaring no pain after 7 d. According to this study’s findings, PP in the laser group was significantly lower than that in the control group 24 h after the first visit. Arslan et al. [34] reported that laser use for intra-canal disinfection reduced PP after the first visit with the application of Ca(OH)_2_, similar to the findings of this study. Furthermore, some other studies reported significantly reduced PP levels in a laser-treated group in single-visit RCT [23, 31]. The root canal obturation procedure can be a risk factor for PP. The results of the aforementioned studies are in line with the findings of the present study on PP levels after root canal obturation. In this study, the laser group tended to have significantly reduced levels of PP both after the application of Ca(OH)_2_ and obturation. Based on this study’s findings, the null hypothesis was rejected. The levels of PP in the present study were generally low in both groups. None of the patients experienced swelling or severe PP that required emergency treatment.

In general, analgesic use is associated with the level of pain. In this study, the patients were advised to use ibuprofen if they experienced high levels of pain. As ibuprofen has dose-dependent activity, 600 mg was recommended for severe pain. The analgesic effect of ibuprofen disappears after 8 h [50]. Thus, analgesic intake 8 h post-treatment did not compromise the evaluation of PP 24 h post-treatment in this study. In the present study, there was no analgesic intake in the laser group. The patients in the control group recorded analgesic intake 8 h and 24 h after the first visit only. The between-group difference in analgesic intake supports the use of laser diode treatment in RCT. Likewise, Arslan et al. [34] and Sen et al. [23] reported that analgesic use in laser groups was significantly lower than that in groups where laser treatment was not applied.

Various factors, such as the treatment protocol and case selection, can affect standardization in randomized clinical studies. The fact that pain is a subjective sensation is the major limitation of this study. Patient’s anxiety and comfort levels before and during RCT, in addition to tissue damage that may occur during anaesthesia or rubber-dam application, could possibly give rise to PP. Additionally, patients who reported on the PP levels, being aware which treatment group they assigned to may have influenced the estimation of results, which is also a limitation of the study. Not assessing the bacterial count of the treated teeth to evaluate the antibacterial effectiveness of the diode laser can be considered as another limitation of this study.

## Conclusion

In conclusion, diode laser application following conventional irrigation may reduce PP in single-rooted necrotic teeth with a PAI score of 3 or 4 after RCT performed in two treatment visits. The findings indicate that diode lasers may be used as part of routine RCT, especially in infected cases, to ensure patient comfort. The present in vivo study may contribute to further studies with larger numbers and different case groups using advanced laser applications.

## Supplementary Information

**Additional file 1**. CONSORT 2010 Checklist.

**Additional file 2**. VAS 1st appointment, Patient’s questionnaire.

**Additional file 3**. VAS 2nd appointment, Patient’s questionnaire.

## Data Availability

Data cannot be shared because in the protocol submitted to the Ethics Committee of University of Biruni, the authors confirmed that only researchers of the University of Biruni would have access to the raw data.

## Abbreviations

RCT: Root canal treatment
PP: Post-operative pain
VAS: Visual analogue scale
NaOCI: Sodium hypochlorite
EDTA: Ethylenediaminetetraacetic acid
Ca(OH)_2_: Calcium hydroxide

## References

[1] Sathorn C, Parashos P, Messer H (2008). The prevalence of postoperative pain and flare-up in single-and multiple-visit endodontic treatment: a systematic review. Int Endod J.

[2] Ng YL, Glennon JP, Setchell DJ, Gulabivala K (2004). Prevalence of and factors affecting post-obturation pain in patients undergoing root canal treatment. Int Endod J.

[3] Mattscheck DJ, Law AS, Noblett WC (2001). Retreatment versus initial root canal treatment: factors affecting posttreatment pain. Oral Surg Oral Med Oral Pathol Oral Radiol Endod.

[4] Çiçek E, Koçak MM, Koçak C (2017). Postoperative pain intensity after using different instrumentation techniques: a randomized clinical study. J Appl Oral Sci.

[5] Alves VO (2010). Endodontic flare-ups: a prospective study. Oral Surg Oral Med Oral Pathol Oral Radiol Endod.

[6] Zehnder M (2006). Root canal irrigants. J Endod.

[7] Berutti E, Marini R, Angeretti A (1997). Penetration ability of different irrigants into dentinal tubules. J Endod.

[8] Zou L, Shen Y, Li W (2010). Penetration of sodium hypochlorite into dentin. J Endod.

[9] Goldman M, Goldman LB, Cavaleri R, Bogis J, Lin PS (1982). The efficacy of several endodontic irrigating solutions: a scanning electron microscopic study. Part 2. J Endod.

[10] Yamada RS, Armas A, Goldman M, Lin PS (1983). A scanning electron microscopic comparison of a high volume final flush with several irrigating solutions: part 3. J Endod.

[11] Baumgartner JC, Mader CL (1987). A scanning electron microscopic evaluation of four root canal irrigation regimens. J Endod.

[12] Bystrom A, Sundqvist G (1985). The antibacterial action of sodium hypochlorite and EDTA in 60 cases of endodontic therapy. Int Endod J.

[13] Hülsmann M, Hahn W (2000). Complications during root canal irrigation—literature review and case reports. Int Endod J.

[14] Seltzer S, Naidorf IJ (2004). Flare-ups in endodontics. I. Etiological factors. J Endod.

[15] Olivi G, Crippa R, Iaria G (2011). Lasers in endodontics Part I. J Laser.

[16] Kumar Rai V, Tabassum S, Zafar S (2015). Lasers in endodontics. Int J Oral Care Res.

[17] Asnaashari M, Ebad LT, Shojaeian S (2016). Comparison of antibacterial effects of 810 and 980-nanometer diode lasers on Enterococcus faecalis in the root canal system—an in vitro study. Laser Ther.

[18] Lopez-Jimenez L, Arnabat-Dominguez J, Vinas M (2015). Atomic force microscopy visualization of injuries in *Enterococcus faecalis* surface caused by Er, Cr:YSGG and diode lasers. Med Oral Patol Oral Cir Bucal.

[19] Gutknecht N, Franzen R, Schippers M (2004). Bactericidal effect of a 980-nm diode laser in the root canal wall dentin of bovine teeth. J Clin Laser Med Surg.

[20] Pirnat S, Lukac M, Ihan A (2011). Study of the direct bactericidal effect of Nd:YAG and diode laser parameters used in endodontics on pigmented and nonpigmented bacteria. Lasers Med Sci.

[21] Schoop U, Kluger W, Dervisbegovic S (2006). Innovative wavelengths in endodontic treatment. Lasers Surg Med.

[22] Koba K, Kimura Y, Matsumoto K (1999). Post-operative symptoms and healing after endodontic treatment of infected teeth using pulsed Nd:YAG laser. Endod Dent Traumatol.

[23] Genç Şen O, Kaya M (2019). Effect of root canal disinfection with a diode laser on postoperative pain after endodontic retreatment. Photomed Laser Surg.

[24] Arias A, Azabal M, Hidalgo JJ, José C (2009). Relationship between postendodontic pain, tooth diagnostic factors, and apical patency. J Endod.

[25] Aşçı SK (2014). Endodonti.

[26] Siqueira JF, Rocas IN, Favieri A (2002). Incidence of postoperative pain after intracanal procedures based on an antimicrobial strategy. J Endod.

[27] Yoldaş O, Topuz A, İşçi AS, Öztunç H (2004). Postoperative pain after endodontic retreatment: single-versus two-visit treatment. Oral Surg Oral Med Oral Pathol Oral Radiol Endod.

[28] Ramamoorthi S, Nivedhitha MS, Divyanand MJ (2015). Comparative evaluation of postoperative pain after using endodontic needle and EndoActivator during root canal irrigation: a randomized controlled trial. Aust Endod J.

[29] Tuner J, Hode L (2007). The laser therapy handbook.

[30] Morsy DA, Negm M, Diab A, Ahmed G (2018). Postoperative pain and antibacterial effect of 980 nm diode laser versus conventional endodontic treatment in necrotic teeth with chronic periapical lesions: a randomized control trial. F1000Research.

[31] Dagher J, El Feghali R, Parker S, Benedicenti S, Zogheib C (2019). Postoperative quality of life following conventional endodontic intracanal irrigation compared with laser-activated irrigation: A randomized clinical study. Photobiomodul Photomed Laser Surg.

[32] Gondim E, Setzer FC, Dos Carmo CB, Kim S (2010). Postoperative pain after the application of two different irrigation devices in a prospective randomized clinical trial. J Endod.

[33] Ramamoorthi S, Nivedhitha MS, Divyanand MJ (2015). Comparative evaluation of postoperative pain after using endodontic needle and EndoActivator during root canal irrigation: a randomised controlled trial. Aust Endod J.

[34] Arslan H, Doğanay E, Karatas E, Ünlü MA, Ahmed HMA (2017). Effect of low-level laser therapy on postoperative pain after root canal retreatment: a preliminary placebo-controlled, triple-blind, randomized clinical trial. J Endod.

[35] Parirokh M, Sadr S, Nakhaee N, Abbott PV, Manochehrifar H (2014). Comparison between prescription of regular or on-demand ibuprofen on postoperative pain after single-visit root canal treatment of teeth with irreversible pulpitis. J Endod.

[36] Topçuoğlu HS, Topçuoğlu G, Arslan H (2018). The effect of different irrigation agitation techniques on postoperative pain in mandibular molar teeth with symptomatic irreversible pulpitis: a randomized clinical trial. J Endod.

[37] Vera J, Ochoa J, Romero M (2018). Intracanal cryotherapy reduces postoperative pain in teeth with symptomatic apical periodontitis: a randomized multicenter clinical trial. J Endod.

[38] Pirnat S (2007). Versatility of an 810-nm diode laser in dentistry: An overview. J Laser Health Acad.

[39] Maturo P, Perugia C (2013). Docimo R Versatility of an 810 Nm Diode laser in pediatric dentistry. Int J Clin Dent.

[40] Pawar S, Pujar M, Makandar S (2014). Postendodontic treatment pain management with low-level laser therapy. J Dent Lasers.

[41] Bjordal JM, Johnson MI, Iversen V (2006). Low-level laser therapy in acute pain: a systematic review of possible mechanisms of action and clinical effects in randomized placebo-controlled trials. Photomed Laser Surg.

[42] Silva Garcez A, Núñez SC, Lage-Marques JL (2006). Efficiency of NaOCl and laser-assisted photosensitization on the reduction of Enterococcus faecalis in vitro. Oral Surg Oral Med Oral Pathol Oral Radiol Endod.

[43] da Costa RA, Nogueira GE, Antoniazzi JH (2007). Effects of diode laser (810 nm) irradiation on root canal walls: thermographic and morphological studies. J Endod.

[44] Gutknecht N, Franzen R, Meister J (2005). Temperature evolution on human teeth root surface after diode laser assisted endodontic treatment. Lasers Med Sci.

[45] Alfredo E, Marchesan MA, Sousa-Neto MD (2008). Temperature variation at the external root surface during 980- nm diode laser irradiation in the root canal. J Dent.

[46] Gutknecht N (2008). Lasers in endodontics. J Laser Health Acad.

[47] Ali SG, Mulay S, Palekar A (2012). Prevalence of and factors affecting post-obturation pain following single visit root canal treatment in Indian population: a prospective, randomized clinical trial. Contemp Clin Dent.

[48] Ng YL, Glennon JP, Setchell DJ, Gulabivala K (2004). Prevalence of and factors affecting postobturation pain in patients undergoing root canal treatment. Int Endod J.

[49] Harrison JW, Baumgartner JC, Svec TA (1983). Incidence of pain associated with clinical factors during and after root canal therapy. Part 2. Postobturation pain. J Endod.

[50] Seltzer S (2004). Pain in endodontics. 1986. J Endod.

