# Supplementary material for: Effect of a 980-nm diode laser on post-operative pain after endodontic treatment in teeth with apical periodontitis: a randomized clinical trial
Source: BMC Oral Health. 2021 Jan 22;21:41. doi: 10.1186/s12903-021-01401-w (PMC7821509; doi:10.1186/s12903-021-01401-w)
Supplement: Supplementary file 2 — Additional file 2. VAS 1st appointment, Patient’s questionnaire. [file 12903_2021_1401_MOESM2_ESM.docx]

**Please mark your pain levels and analgesic intake status after the first appointment of root canal theraphy:**

(0:No / 1,2,3: Mild / 4,5,6: Moderate / 7-8-9-10: Severe)

**8 hours after treatment:**

0 - 1 - 2 - 3 - 4 - 5 - 6 - 7 - 8 - 9 - 10

**Did you take any analgesics?**

Yes □ No □

**24 hours after treatment:**

0 - 1 - 2 - 3 - 4 - 5 - 6 - 7 - 8 - 9 - 10

**Did you take any analgesics?**

Yes □ No □

**48 hours after treatment:**

0 - 1 - 2 - 3 - 4 - 5 - 6 - 7 - 8 - 9 - 10

**Did you take any analgesics?**

Yes □ No □

**7 days after treatment:**

0 - 1 - 2 - 3 - 4 - 5 - 6 - 7 - 8 - 9 - 10

**Did you take any analgesics?**

Yes □ No □
